# The impact of residual ridge morphology on the masticatory performance of complete denture wearers

**DOI:** 10.1016/j.heliyon.2023.e16238

**Published:** 2023-05-13

**Authors:** M.T. Sta Maria, Yoko Hasegawa, Pinta Marito, Tasuku Yoshimoto, Simonne Salazar, Kazuhiro Hori, Takahiro Ono

**Affiliations:** a Division of Comprehensive Prosthodontics, Faculty of Dentistry, Niigata University Graduate School of Medical and Dental Sciences, Niigata, Japan; b Department of Prosthodontics, College of Dentistry, Manila Central University, Caloocan, Philippines; c Department of Prosthodontics, Faculty of Dentistry, Universitas Indonesia, Jakarta, Indonesia; d Department of Prosthodontics, Faculty of Dentistry, Centro Escolar University, Makati, Philippines; e Department of Geriatric Dentistry, Faculty of Dentistry, Osaka Dental University Osaka, Japan

**Keywords:** Residual ridge, Masticatory performance, Gummy jelly, Complete dentures, Mastication

## Abstract

**Statement of the problem:**

Morphology of the residual ridge (RR) is expected to influence the masticatory performance (MP) of complete denture (CD) patients, but considerable details of this relationship are unknown.

**Purpose:**

We aimed to investigate the association between the objective MP and RR morphology of CD wearers and other contributory factors affecting their MP.

**Materials and methods:**

Sixty-five patients with well-fitting upper and lower CDs with no complaints of pain were enrolled. The objective MP was measured using test gummy jelly and a fully automated measuring device. The RR form was divided into U-type, V-type, I-intermediate, and F-Flat, then combinations of upper and lower RR forms (combined RR) were classified. The height was measured using CD's denture basal surface replicas, while occlusal contact of CDs was assessed using a tooth contact analysis system. The relationship between surveyed factors and MP was evaluated using Spearman's rank correlation, Kruskal-Wallis test, generalized linear regression, and analysis of covariance.

**Results:**

Participants with F–F and V–F combined RR forms had the lowest MP, while those with U–U and U–I forms had the highest MP, regardless of RR height. Participants with low RR height had the lowest MP, and those with high RR height had the highest MP, regardless of RR form. The analysis of covariance revealed that mandibular RR height, combined RR forms, and total occlusal contact area significantly affected the MP.

**Conclusions:**

Our findings confirmed that the mandibular RR height, RR form combinations, and occlusal contact influence the MP of CD wearers.

*ClinicalImplications:*

The MP of CD wearers varied, depending on the height and form of the RR, as well as the occlusal contact area of the CDs. The results of this manuscript show that the morphology of the denture bearing area and the occlusion of the CDs are essential factors in predicting the treatment outcome of CD wearers. This allows the clinician to fabricate a complete denture with the denture basal surfaces adjusted and occlusion provided according to the patient. CD patients can be educated on how to chew to improve MP based on their own RR morphology.

## 1 Introduction

The number of associated health problems increases drastically as aging progresses [1], eventually leading to a decline in the oral status among older people [2]. The reduced masticatory function associated with aging [[1], [2], [3]], cause several health problems, such as nutritional deficiencies [4], metabolic syndrome [5], hypertension [6], and stroke [7]. In Japan, the proportion of removable denture (hereinafter abbreviated as “denture”) wearers increases significantly with age, with about half of those ≥85 years old wearing complete dentures (CDs) [8]. Studies have reported that CD wearers have more difficulty chewing hard foods than those with natural dentition [9,10], with their masticatory performance (MP) being around 50% of those with natural dentition [[10], [11], [12], [13], [14], [15]]. CD wearers chew longer, increasing the number of chewing cycles at a decreased masticatory rate and swallowing coarser food particles [16], eventually influencing their general health, increasing the risk of physical frailty, cognitive decline, and reduced quality of life [10]. Therefore, it is important to maintain and manage their masticatory function so that CD wearers do not suffer from malnutrition.

The MP of denture wearers is influenced by many factors, such as tooth loss [13,17,18], residual ridge [1,9,13,[18], [19], [20], [21]], maximum bite force [[15], [17], [22], [23]], the tongue [[22], [24]], and lip function [2], salivary secretion [15,25,26], previous experience with dentures [15], and denture stability, and retention [9,15,21,27,28]. Among those factors, poor residual ridge (RR) morphology has always been a primary concern for clinicians hoping to achieve successful CD treatment because RR height and form are essential to support the denture and prevent dislodgment [21]. RR resorption leads to a decrease in the RR height [1,29], and the result is not only a decrease in the denture supporting areas but also a decrease in bracing and salivary retention [19]. In addition, the morphology of the RR is complex [1], and it has been pointed out that the morphology has a significant influence on the functional performance of dentures because the morphology of the RR is related to the stress distribution at the denture bearing areas [30]. In addition, in cases of upper and lower CD, the combination of RR morphology is expected to affect denture stability and, consequently, MP, but we have not found any studies reporting on this. The relationship between RR morphology and MP in CD patients has been shown in several papers [9,15,18,31,32]. On the other hand, in these reports, no studies examined contributory factors other than RR morphology that affect MP.

Therefore, for CD wearers, our study aimed to investigate the association between the objective MP and RR morphology, focusing not only on its height and form but also on the combination of upper and lower RR. In addition, we also clarified the factors that affect the MP of CD wearers by multivariate analysis considering other factors aside from RR morphology.

## 2 Materials and methods

This observational cross-sectional study was conducted after applying to and receiving approval from the Niigata University Ethics Committee (#28-R42-28). After a thorough explanation of the study, oral and written informed consent was obtained from the participants.

### 2.1 Participants

The participants of this study were CD wearers who visited the Department of Removable Prosthodontics from June 2015 to July 2021. This study selected participants according to the following inclusion criteria: patients with an edentulous maxilla and mandible; currently wearing well-adapted upper and lower CDs; no complaints of pain or any functional disturbances; no known history of temporomandibular joint disorders; and able to understand and respond appropriately to a questionnaire. This study excluded individuals with maxillofacial defects and overdentures.

### 2.2 Fabrication of a CD replica

**Fig. 1:**
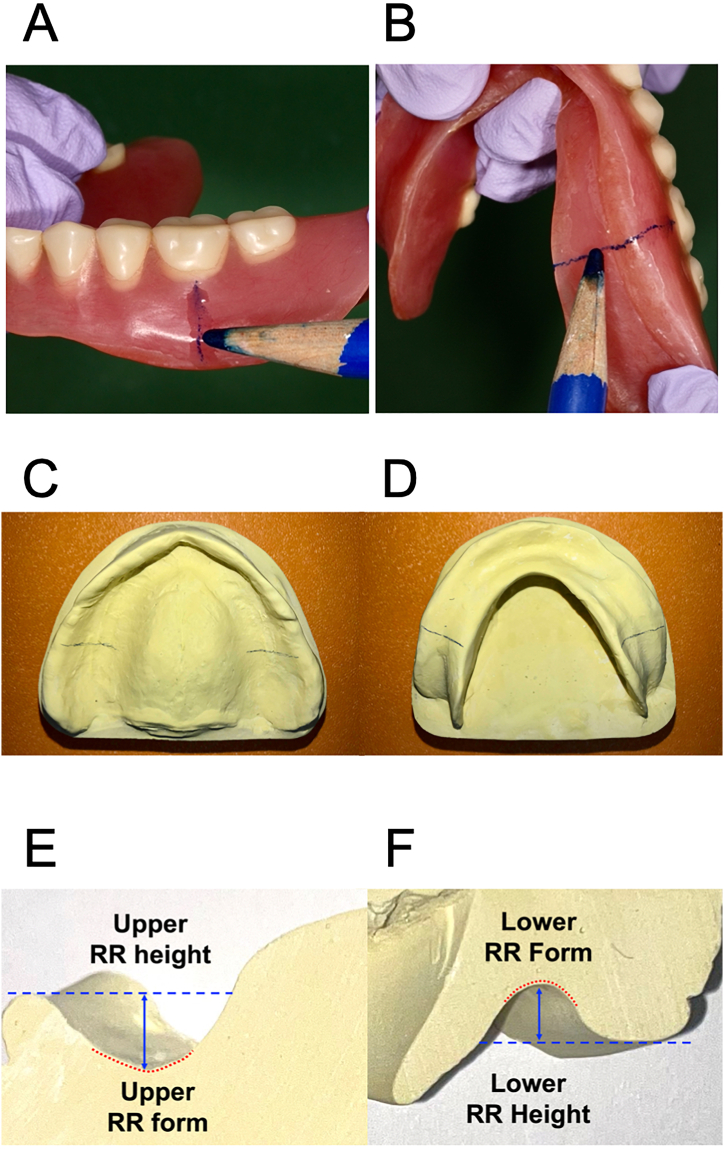
Procedures for assessing RR form and height. A, Marking the center of the first molar location on the polished surface of the mandibular CD. B, marking the center of the first molar location on the denture basal surface of the CD. C, maxillary CD replica models marked on the location of the first molars. D, mandibular CD replica model marked on the location of the first molars. E, cross-sectional view of the maxillary RR. F, cross-sectional view of the mandibular RR.

Before all assessments, the adaptation of the CDs to the underlying tissues under static or dynamic conditions was checked using a pressure-indicating paste made of silicone (Fit Checker; GC Dental Corporation). We also confirmed that the patients did not feel pain or discomfort in the denture bearing areas when the CDs were worn. Before the assessment of the RR form and height, the maxillary and mandibular CDs of each subject were marked with a copying pencil (B + U 1925 Jolly: Austria Kopierstift, Brevillier Urban & Sachs GmbH & Co KG) at the location of the first molars as shown in Fig. 1A and B. Replicas of the polished and denture basal surfaces of the CDs were fabricated using alginate impression material (Aroma Fine Plus, GC Dental Corporation, Tokyo, Japan) and dental plaster (New Plastone II, GC Dental Corporation). Since the marked location of the bilateral molars was marked with a copy pencil, the markings were transferred instantly onto the denture basal surfaces of the CD models ((Fig. 1C and D). When the markings were deeemed accurate, the models were trimmed cross-sectionally at the marked locations of the first molars. The RR height and form were evaluated from this cast (Fig. 1E and F).

### 2.3 The assessment of RR morphology

The term “morphology” used in this study was the collective term for the “form” and “height” of the residual ridge [33]. The term “form” was defined as the shape of the RR [34] or the cross-sectional evaluation of the RR [35], while “height” means the vertical evaluation of the RR [36].

### 2.4 The assessment of the RR form and the combination of the maxillo-mandibular form

**Fig. 2:**
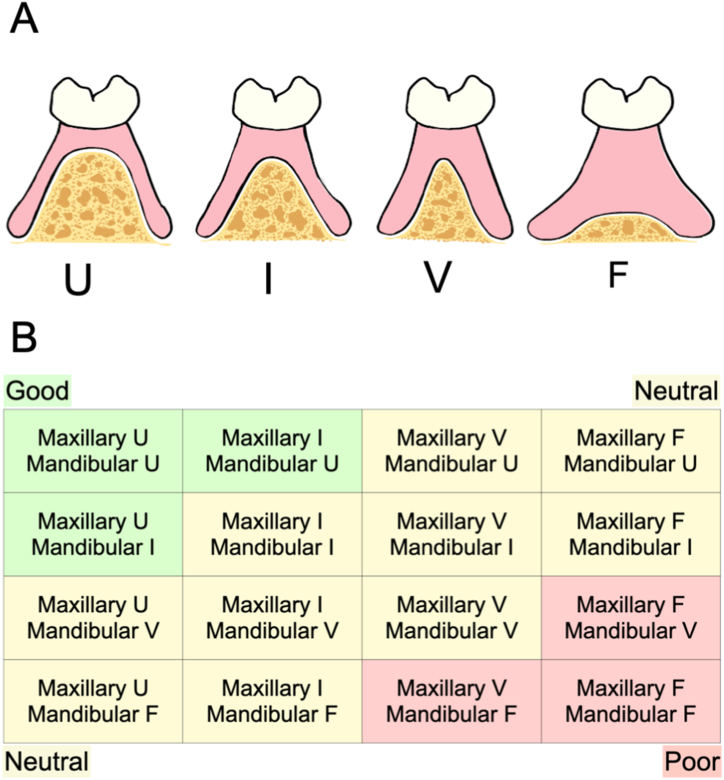
Assessment of the RR form. A, Classification of the RR form of the maxilla and mandible according to Kapur: U-type; I = intermediate (between U and V); V-type; and F = Flat. B, Classification of the combined maxillary and mandibular RR forms.

The RR forms of the maxilla and mandible were first checked intraorally by two prosthodontics specialists certified by the Japan Prosthodontic Society. Furthermore, using the trimmed replica models of the maxillary and mandibular CDs, the maxillary and mandibular RR forms were evaluated at the marked location of the first molar using the following criteria: 1 = U-type; 2 = Intermediate (between U and V); 3 = V-type; and 4 = Flat according to Kapur's [37] index (Fig. 2A). Kapur's index is used universally for the clinical evaluation of denture bearing tissues, such as tissue resiliency, location of border tissue attachment, and the shape of the RR [37]. The RR forms were assessed cross-sectionally according to Kapur's index [37]. It is generically used to evaluate the degree of RR resorption. For this reason, it was used in this study. The left and right sides were assessed for each maxilla and mandible RR form, and the side with the poorer RR form was selected.

According to the maxillary and mandibular RR forms, the participants were classified into the “good” group, the “neutral” group, and the “poor” group (Fig. 2B). For example, the RR form was considered “good” if the maxillary and mandibular RR were both U-type, or if the maxillary RR is intermediate and the mandibular RR was U-type, and if the maxillary RR was U-type and the mandibular RR is intermediate. The RR form was considered “bad” if the maxillary and mandibular RR were both flat, or if the maxillary RR was V-type and the mandibular RR was flat, and if the maxillary RR was flat and the mandibular RR was V-type. RR with combinations other than those mentioned was considered “neutral.”

### 2.5 The assessment of the RR height

The RR heights of the maxilla and mandible were measured directly on the CDs and the replica models of the CDs (Fig. 1E and F) using a digital depth gauge (Digital Depth Gauge mini, product no. 19305; Shinwa Rules Co., Ltd.). The RR height was evaluated at the marked location of the first molar on the denture basal surface of the CD model using the buccal flange as the reference point. The left and right sides of the maxillary and mandibular CDs were also measured separately. Then the averages of the left and right sides of the two assessments were selected for comparison. When the two prosthodontic specialists disagreed in the evaluation, the decision of a third evaluator resolved the disagreement.

The RR height was further classified into three groups: high group (RR height greater than the mean value + 1 standard deviation [SD] of all patients); middle group (RR height ranged from the mean value – 1 SD to the mean value + 1 SD of all patients); and low group (RR height <1 SD of all patients).

### 2.6 The MP evaluation

**Fig. 3:**
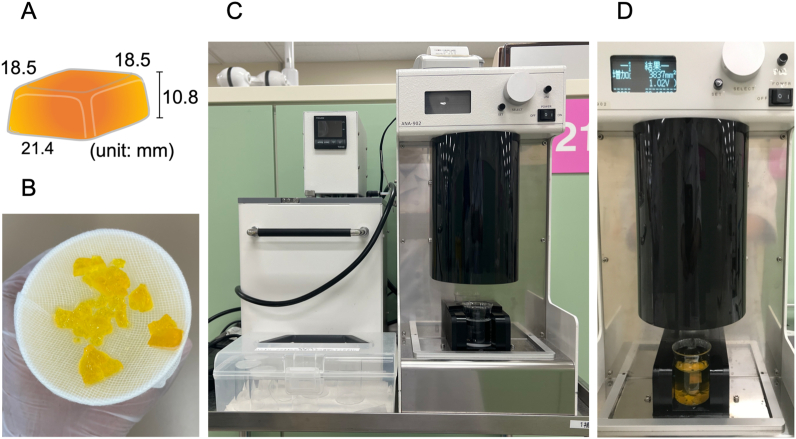
MP evaluation. A, piece of test gummy jelly with measurements. B, comminuted gummy jellies collected in the receptacle. C, fully automated measuring device. D, fully automated score displayed after the analysis of the comminuted gummy jellies.

The MP was evaluated using a fully automated measuring device (Fig. 3C and D) developed by Nokubi et al. [38,39] Initially, the participants were asked to chew a piece of test gummy jelly as seen in Fig. 3A (dimensions: 21.4 × 18.5 × 10.8 mm; weight: 5.5 g; UHA Mikakuto), 30 times on their preferred chewing side with conscious swallowing restrictions. After chewing, the participants were asked to expectorate the chewed gummy jelly onto a prepared receptacle covered with gauze (Fig. 3B). The comminuted gummy jellies were then collected, rinsed with running water (to remove saliva adhering to the surface), and transferred to a clear cell that was placed in the fully automated measuring device (Tokyo, Photoelectric). The degree of comminution was calculated based on the concentration of β-carotene dissolved from the comminuted pieces of gummy jelly in a 25 ml aqueous solution and expressed as the increase in the surface area (mm^2^) after calculation (Fig. 3D). This measurement was used as the value for the MP [14,38,40,41].

### 2.7 The occlusal contact area analysis

**Fig. 4:**
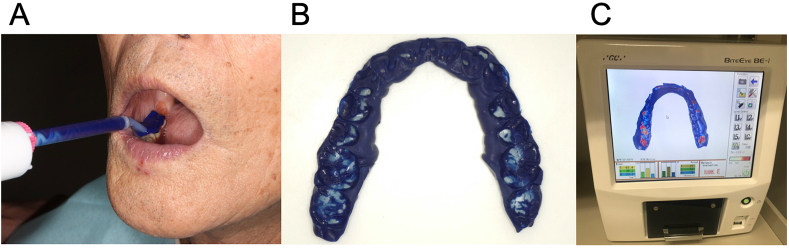
Occlusal contact area analysis. A, Occlusal contact inspection material dispensed inside the patient's mouth. B, trimmed material. C, digitized and analyzed material in the tooth contact analyzing machine.

The occlusal contact areas of the CDs were also checked by taking a bite impression using occlusal contact inspection material (GC Blue Silicone; GC Dental Corporation). The participants were asked to open their mouths. Then the silicone was laid over the occlusal surfaces of the mandibular artificial teeth (Fig. 4A). The participants were asked to close their mouths naturally. They were instructed to maintain the occlusal contact between the upper and lower CDs for 90 s until it was set. The set material was removed from the subject's mouth, disinfected, trimmed (Fig. 4B), and scanned using a tooth contact analyzer (BiteEye BE-I; GC Dental Corporation). Impressions obtained by the bite registration were photographed under upper and lower lighting. The impression's contact area and thickness were analyzed from the light transmittance, and the image was synthesized and displayed (Fig. 4C). The results of the occlusal contact analysis were indicated by color, area, and score. The thickness of the impression body was displayed in different color gradients ranging from green (180–200 μm), yellow (60–69 μm), to dark red (0–4 μm). Occlusal contact was defined as the area with <50 μm or the areas marked with red. However, in this study, only the total occlusal contact area (mm^2^) and point total (number) were used for the comparison.

### 2.8 Statistical analyses

The normality of the data distribution was checked using the Shapiro-Wilk test. When the data were non-normally distributed, square root or logarithmic transformations were performed. Afterward, the data's normality was rechecked and was still found to be non-normally distributed. Hence, non-parametric methods were used for all statistical analyses. Intra-class coefficient (ICC) [[42], [43], [44]] analyses were performed to verify the inter-rater reliability of the evaluators of the RR height measurements and between direct CD measurement and the CD replica models. Non-parametric tests, including Kruskal-Wallis and Bonferroni-adjusted Mann-Whitney-U tests, were used to compare the MP of each group of categorized items. For the group bias analysis, comparisons were made between the maxillary and mandibular RR form and height groups using Pearson's chi-square tests. Spearman's rank correlation coefficient was used to assess the associations between the independent variables and the MP of CD wearers. A generalized linear model was used to identify the RR form most strongly affecting the MP. The MP was analyzed using an analysis of covariance (ANCOVA) to determine the variables that substantially affected the MP of CD wearers. The ANCOVA models included: 1. variables that were significantly related to MP as confirmed by the Kruskal-Wallis test or Spearman's rank correlation coefficient as the simple main effect, 2. interactions with the simple main effect, 3. the interaction term was added when there was a significant interaction between the main effects, after adjusting for the age and duration of CD wearing.

The statistical analyses were performed using the IBM SPSS Statistics software program, Version 25 (IBM), and the probability values of less than 5% were considered significant.

## 3 Results

### 3.1 Subject characteristics

The characteristics of the participants of this study are shown in Table 1. In total, 65 completely edentulous individuals were assessed for participation in this study (mean age: 77.6 ± 1.0 years old). Among the participants, 63% were female, and the average duration of CD use was about two years (median: 329 ± 97.6 days; mean duration: 706.5 days). An extensive range of RR form and RR height was found in the maxillary and mandibular arches of the participants in this study. First, 27.7% of participants were determined to have a flat maxillary RR form, while half had a flat mandibular RR form. Second, when the maxillary and mandibular RR forms were combined, 26.2% of participants had poor RR forms, while 52.3% were classified as “neutral.” Lastly, 72.3% of maxillary and 61.5% of mandibular RR heights were classified as “middle” among the subjects.

**Table 1:** Characteristics of participants.

**Factors**	**Participants (N = 65)**
***Personal Factors***	
Age (years)	77.62 ± 1.04
Gender	
Male	24 (36.9)
Female	41 (63.1)
Duration of denture wearing (days)	706.5 ± 97.6
Masticatory Performance (mm^2^)	1534.90 ± 152.69
***Denture Condition***	
Occlusal Contact	
Area Total (0–90.8 mm^2^)	24.43 ± 2.62
Point Total (2–122)	28.52 ± 2.24
***Residual Ridge Assessment***	
**RR Form**	
***Maxillary RR Form***	
U-type	28 (43.1)
I-Intermediate	15 (23.1)
V-type	4 (6.2)
F-Flat	18 (27.7)
***Mandibular RR Form***	
U-type	5 (7.7)
I-Intermediate	18 (27.7)
V-type	10 (15.4)
F-Flat	32 (49.2)
***Combined RR Form***	
good (U&U, U&I)	14 (21.5)
neutral	34 (52.3)
poor (F&F, V&F)	17 (26.2)
**RR Height**	
***Maxillary RR Height (mm)***	7.76 ± 0.26
High	6 (9.2)
Middle	47 (72.3)
Low	6 (9.2)
Unknown	6 (9.2)
***Mandibular RR Height (mm)***	4.50 ± 0.33
High	9 (13.8)
Middle	40 (61.5)
Low	10 (15.4)
Unknown	6 (9.2)

Data are presented as the mean ± standard error or the number of participants (%).

Masticatory performance: the increase in the surface area of the masticated gummy jelly (mm^2^). RR: residual ridge. RR Form: The RR form was considered good if the maxillary and mandibular RR were both U-type, or if the maxillary RR was intermediate and the mandibular RR was U-type, and if the maxillary RR was U-type and the mandibular RR was intermediate. The ridge form was considered poor if the maxillary and mandibular RR were flat, or if the maxillary RR was V-type and the mandibular RR was flat, and if the maxillary RR was flat and the mandibular RR was V-type. Combinations other than those mentioned were considered neutral. Maxillary and mandibular RR height: The RR height values were classified into three groups: high group (RR height was greater than the mean value + 1 SD of all patients); middle group (RR height ranged from the mean value minus 1 SD to the mean value + 1 SD of all patients); and the low group (RR height was <1 SD of all patients).

Of the 65 cases presented, direct measurements of the denture and the CD replica models were performed on 37 cases. Since there were 28 cases without CD replica model measurements, we verified the reliability of the measurements. The ICC between the CD replicas and the direct measurement of the denture was 0.827 (P < 0.001). This result confirmed that the measurements were reliable and that the CD replica model measurements could be used to evaluate the RR height. Since the measured values of the CD replica models were like those obtained by measuring the CDs directly, the values measured directly on the CDs were used when the replica model could not be fabricated. Of the 28 cases without CD replica model measurements, there were six cases without records of RR height measurement but with records for RR form and MP. Therefore, these participants were still included in the study, and unmeasured RR height data were treated as missing values.

The ICC of the two evaluators who used the same models to assess the RR height was 0.744 (P < 0.001), indicating that the evaluation was reliable.

### 3.2 MP and the surveyed items

Comparisons of MP by the surveyed items are shown in Table 2. The findings revealed that there was no difference in the MP between genders. Although there were no significant differences in the type of maxillary or mandibular RR forms, the MP of participants did vary depending on the type of RR form and the affected jaw. The poor group (F&F, V&F) exhibited the lowest MP when the maxillary and mandibular RR forms were combined. In the neutral group, the combination of maxillary F and mandibular U had an MP of 861.7 ± 390.7 mm^2^ (mean ± standard error), which was even lower than the combination of maxillary U and mandibular F (1466.7 ± 949.1 mm^2^). In contrast, the MP of maxillary F and mandibular I combinations was higher (2343.9 ± 2064.9 mm^2^) than that of maxillary I and mandibular F (1276.3 ± 1106.5 mm^2^).

**Table 2:** The relationship between masticatory performance and residual ridge morphology: A comparison by each category.

**Factors**	**Masticatory Performance (mm**^**2**^**)**	**p-value**	**Two-group Comparison**
**Gender**		0.28	N.S.
Male	1756.11 ± 262.52		
Female	1405.40 ± 186.56		
**RR Form**			
***Maxillary RR Form***		0.07	N.S.
U-type	2030.43 ± 248.41		
I-Intermediate	1196.73 ± 255.85		
V-type	822.35 ± 124.68		
F-Flat	1204.21 ± 278.95		
***Mandibular RR Form***		0.17	N.S.
U-type	2415.68 ± 668.18		
I-Intermediate	1801.46 ± 326.68		
V-type	1611.97 ± 435.70		
F-Flat	1223.25 ± 173.86		
Combined RR Form*		0.012*	a^†^
Good	2434.09 ± 367.35		
Neutral	1419.86 ± 187.50		
Poor	1024.46 ± 248.36		
**RR Height**			
***Maxillary RR Height***		0.76	N.S.
High	2063.72 ± 616.92		
Middle	1558.89 ± 173.07		
Low	1338.03 ± 622.59		
Unknown	1015.00 ± 432.84		
Mandibular RR Height*		0.007*	b^†^, c^†^
High	2044.10 ± 491.26		
Middle	1708.82 ± 192.18		
Low	692.88 ± 214.00		
Unknown	1015.00 ± 432.84		

Data are presented as the mean ± standard error.

RR: Residual ridge. RR Form: U-type; Intermediate (between U and V): V-type; and Flat were decided according to Kapur's index [37]. Combined RR Form: According to the maxillary and mandibular RR forms, the participants were classified into three groups: Good group, Neutral group, and Poor group (Fig. 2B). Maxillary and mandibular RR height: The RR height values were classified into three groups: high group (RR height was greater than the mean value + 1 SD of all patients); middle group (RR height ranged from the mean value minus 1 SD to the mean value + 1 SD of all patients); and the low group (RR height was <1 SD of all patients).

*: Significant statistical differences were seen using the Kruskal-Wallis Test (p < 0.05).

^†^: Significant statistical differences were seen using the Mann-Whitney *U* Test (p < 0.05).

Two-group comparison: Bonferroni-adjusted Mann-Whitney-U tests were performed.

a: Significant statistical differences were seen between the poor and good groups; b: Significant statistical differences were seen between the low and middle groups; c: Significant statistical differences were seen between the high and low groups.

Although significant statistical differences were found in the middle and high groups of mandibular RR height compared to the low group (p = 0.007), there was no significant difference between the groups of maxillary RR height. Furthermore, participants with a low mandibular RR height had the lowest MP among all groups. When we examined the relationship between RR height and RR form, Spearman's rank correlation coefficient revealed that both were significantly associated at p < 0.01 (maxillary r = 0.364; mandibular r = 0.616). In other words, if a participant's RR height was low, their RR form was also low.

Table 3 shows the factors significantly associated with the MP of participants in this study. Age, however, was found to have no significant association with MP. The RR height and occlusal contact point total were found to be significantly correlated with the MP of participants at p < 0.01, while the total occlusal contact area and duration of wearing the CD significantly correlated with the MP at p < 0.05.

**Table 3:** The correlation between the masticatory performance and the numerical evaluation items of complete denture wearers.

**Factors**	**r**	**p-value**
Age (years)	−0.148	0.238
Duration of denture wearing (days)*	0.268*	0.031
*Occlusal Contact*		
Point Total (2–122)^a^	0.346^a^	0.006
Area Total (0–90.8 mm^2^)*	0.291*	0.022
Maxillary RR Height (mm)^a^	0.359^a^	0.003
Mandibular RR Height (mm)^a^	0.349^a^	0.004

r: Spearman's rank correlation coefficient. RR: residual ridge.

a : A significant correlation was noted if the p-value <0.01; *: if p-value <0.05.

### 3.3 Morphological factors affecting the MP

Since the MP of the participants varied depending on the type of RR form, we used a generalized linear regression analysis to identify the RR form that most strongly influenced the MP (Table 4). The results showed that, regardless of the affected jaw, the flat RR form had a more substantial effect on the MP of CD wearers than the U-type RR form. Regarding the maxillary RR morphology, it was shown that the MP decreased significantly as the resorption and flattening of the RR progressed. The mandibular morphology exhibited the same tendency as the maxilla, although the model effect was insignificant. Furthermore, when the RR forms were combined, the poor group (F&F, V&F) had a strong effect than the good group (U&U, U&I). The findings revealed that the MP decreased significantly in the order of Good, Neutral, and Poor.

**Table 4:** The influence of residual ridge form on the masticatory performance of complete denture wearers.

				**Wald Confidence Interval**		
**Explanatory variables**	**B**	**standard error**	**Wald chi-square**	**Lower**	**Upper**	***P*****-value**
***Maxillary RR Form***						
U–type	ref	–	–			
I-Intermediate^a^	−833.7	364.59	5.23	−1548.29	−119.11	0.022
V–type^a^	−1208.09	609.07	3.93	−2401.83	−14.34	0.047
Flat^a^	−826.23	344.24	5.76	−1500.93	−151.53	0.016
intercept	2030.44	215.34	88.91	1608.38	2452.49	<0.001
***Mandibular RR Form***						
U–type	ref	–	–			
I-Intermediate	−614.22	590.4	1.08	−1771.39	542.94	0.298
V-type	−803.71	639.68	1.58	−2057.47	450.05	0.209
Flat^a^	−1192.43	561.62	4.51	−2293.19	−91.67	0.034
intercept	2415.68	522.3	21.39	1391.99	3439.37	<0.001
***Combined RR Form***						
good (U&U, U&I)	ref	–	–			
neutral^a^	−1014.23	354.02	8.21	−1708.1	−320.37	0.004
poor (F&F, V&F)^a^	−1409.63	402.35	12.27	−2198.22	−621.05	<0.001
intercept	2434.09	297.95	66.74	1850.12	3018.07	<0.001

The generalized linear regression analysis for the evaluation of each residual ridge form assessment.

RR: residual ridge. Dependent variable: masticatory performance (mm^2^), explanatory variables: type of RR form (Fig. 2A and B). B: partial regression coefficient. Maxillary RR Form model: Wald chi-square = 9.04, P = 0.021. Mandibular RR Form model: Wald chi-square = 6.10, P = 0.107. Combined RR Form model: Wald chi-square = 13.03, P = 0.001.

a : Significant if p-value <0.05.

### 3.4 Factors related to the MP

Considering that age, duration of wearing, total occlusal contact area, occlusal point total, RR height, and combined RR forms were found to be significantly associated with MP, as seen in Table 2, Table 3, Table 4, an ANCOVA was performed to identify which of these factors had the most substantial impact on the MP of the participants in this study. The results revealed that significant independent variables with a substantial effect on the MP of CD wearers were mandibular RR height, combined RR forms, and total occlusal contact area (Table 5).

**Table 5:** Factors affecting the masticatory performance of complete denture wearers.

				**Wald Confidence Interval**		
**Explanatory variables**	**B**	**standard error**	**Wald chi-square**	**Lower**	**Upper**	***P*****-value**
Mandibular RR Height (mm)^a^	0.76	0.19	16.66	0.40	1.13	<0.001
Combined RR Form: Good^a^	1.72	0.49	12.17	0.75	2.69	<0.001
Combined RR Form: Poor^a^	1.53	0.47	10.65	0.61	2.45	0.001
Combined RR Form: Neutral^a^	1.59	0.49	10.49	0.63	2.55	0.001
Total occlusal contact area (mm^2^)^a^	0.85	0.35	5.85	0.16	1.54	0.016
Occlusal Point Total	0.43	0.37	1.35	−0.29	1.15	0.246
Age (years)	0.14	0.13	1.10	−0.12	0.40	0.295
Maxillary RR Height (mm)	−0.28	0.31	0.83	−0.89	0.32	0.363
Duration of denture wearing (day)	0.06	0.07	0.75	−0.08	0.20	0.388
Total occlusal contact area × Occlusal Point Total	−0.43	0.24	3.14	−0.90	0.05	0.077
Intercept	.08	0.02		0.06	0.12	

The analysis of covariance (ANCOVA) was performed.

RR: residual ridge. B: partial regression coefficient. Dependent variable; masticatory performance (mm^2^), the simple main effect; factors that were significantly related to the masticatory performance by Kruskal-Wallis or Spearman's rank correlation coefficient (Table 2, Table 3). Interaction term: between total occlusal contact area and occlusal point total.

a : Significant if p-value <0.05.

## 4 Discussion

The primary aim of this study was to evaluate the MP of CD wearers with different types of RR morphology. We observed in this study that the degree of mandibular RR resorption, the combination of maxillary and mandibular RR forms, and the occlusal contact area of CDs significantly influenced the MP of CD wearers. The MP of participants in the current study varied significantly depending on the RR form and height. Furthermore, participants with a low RR height, poor RR forms, and a low total occlusal contact area had a lower MP than others. Our study suggests that the RR morphology and occlusal contact status of the CDs potentially influence the treatment outcome of CDs.

### 4.1 MP and RR morphology

Mastication is an intricate yet vital process in nutritional intake wherein the teeth, tongue, and muscles of mastication work harmoniously to form a bolus suitable for swallowing [4,45]. Masticatory performance is one of the most well-known assessments of mastication, determining an individual's ability to comminute a given test food in a fixed number of chewing cycles (MP) [14]. Previous research has shown that impaired mastication leads to poor eating habits and malnutrition [[46], [47], [48]] and the development of metabolic syndrome [5], which causes cerebrovascular disease [47] in frail older adults. Therefore, it is critical to evaluate MP and investigate factors that influence it to prevent the deterioration of general health.

According to the Japanese Society of Gerodontology [2], an individual may have decreased masticatory function if the evaluation of masticatory ability by gummy jelly was less than score two or less than 1100 ± 135 mm^2^ [13]. The mean MP of the participants in this study was as low as 1534.90 ± 152.69 mm^2^. This suggests that the participants in this study have decreased masticatory function.

In our study, the large percentage of CD wearers with flat RR forms for maxillary (27.7%) and mandibular (49.2%) arches show the need to address issues that would help preserve the residual alveolar ridge because the contour of the RR dictates the denture basal surfaces of the CDs during function and how it affects the MP of patients [49]. RR resorption is an irreversible, cumulative, progressive disease [18] that occurs after tooth extraction and is caused by anatomical, functional, metabolic, and prosthetic factors [19,50,51], but it can be slowed down with the help of an appropriate prosthodontic treatment [1,20]. According to our study, the MP of participants varied depending on the RR form. Surprisingly, participants with a V-type maxillary RR form had lower MP than those with a flat RR. It is possible that a V-type RR form act as a fulcrum during chewing, causing denture instability and pain at denture pressure points which jeopardize the outcome of CD treatment. A study has reported that the mandibular RR shape was significantly associated with the masticatory ability of CD wearers by using a 25-item food intake questionnaire [9]. However, their method of evaluating the RR shape differed from the present study as they quantified the volume and height of each replica of mandibular arches and used it to classify the RR shape into small, moderate, and large basal areas. Another study used a classification system developed by the American College of Prosthodontists (ACP) to classify denture-supporting structures considering bone height using radiographs [31]. Because the measurement of maxillary bone height using radiographs is reported to be unreliable [52], ACP only measured the mandibular bone height and judged the maxillary arch's RR morphology instead. In our study, we performed the measurements on the CD replica, which showed the RR height in real-time for both maxilla and mandibular arches.

Previous studies have also shown that RR morphology and resiliency influenced the retention and stability of conventional CDs [21,31,53]. Retention and stability are said to be determining factors for chewing ability [15].

Meanwhile, when the RR forms of the maxillary and mandibular jaws were combined, the poor group (F&F, V&F) had a stronger effect on the MP than the good group (U&U, U&I), indicating that the MP of the poor group was significantly lower than that of the good group. This was consistent with the study that reported that patients with V-type and flat RR forms have difficulty wearing their dentures [54], consequently affecting their MP. To our knowledge, this was the first study that clarified the relationship between the combined RR form and MP.

When the RR remodels after tooth loss, not only it changes shape but also loses its height, making the maxillary arch appear narrower while the mandibular arch appears wider [37]. The atrophy rate of the mandible in CD wearers is higher than the maxilla due to the unfavorable shape and size of the RR on which mechanical load is applied during chewing [1]. The mandibular RR height is approximately four times lower than the maxilla's, making it less capable of resisting occlusal forces [37]. According to our study, the mandibular RR height significantly influenced the MP of CD wearers. This was supported by the results of the covariance analysis, indicating that the mandibular RR height had the strongest effect on the MP of CD wearers, followed by the combined RR forms and total occlusal contact area. This result was consistent with another study which found that patients with a normal mandibular RR height had a better MP than those with an RR resorption [15]. Their findings suggested that the mandibular RR height was strongly related to mandibular CD retention and stability, causing discomfort during the function and eventually affecting the MP [15]. On the contrary, our findings opposed those of previous studies, which found no significant relationship between RR height using panoramic radiographs and MP using the sieving method with peanuts [31,55].

The present findings also showed that the MP of CD wearers tended to decrease as the resorption and flattening of the RR progressed, which was significantly evident in the maxilla but not in the mandible. In other words, as the maxillary RR form flattens, the MP of CD wearers also decreases. This is probably because those with atrophied maxilla wear heavier dentures to compensate for the lost vertical dimension, resulting in the loss of its peripheral seal and, consequently, denture retention [56]. As for the mandible, it does not necessarily imply that the MP decreases even if the mandibular RR form flattens, as the tongue and the cheeks can stabilize the mandibular denture during chewing [22].

### 4.2 Other factors related to the MP

According to the findings of this study, the occlusal contact area was a factor that had a significant influence on the MP of CD wearers. Our results showed that if the total occlusal contact area was low, the MP of the participants was also low. This concurred with another study that found that denture quality and occlusal support influenced the MP of denture-wearing patients [14]. Mastication was reported to be heavily dependent on the occlusion [13]. Hence, inadequate denture occlusion can significantly influence denture stability and cause tissue trauma, making it difficult for patients to use their dentures [53] successfully. Another study found that an increased occlusal contact area, regardless of the anatomic or non-anatomic posterior tooth form, can improve the MP in CD wearers [28]. However, one of the limitations of our study was that we did not take into account the type of occlusion as a factor that influenced the MP of participants in this study.

One of the factors that were also found to be positively correlated with the MP of participants in this study was the duration of CD wearing. This concurs with the findings of previous studies, which showed that increased MP in denture patients was positively related to a longer period of wearing dentures [15,16,32]. According to one study, patients rehabilitated with new CDs needed more time to adjust to their new dentures to improve their masticatory function [32]. A study has reported that participants with normal mandibular RR have higher MP and satisfaction with their new CDs after installation regardless of the follow-up compared to participants with resorbed RR [15]. This was consistent with the findings of another study, which reported a significant improvement of the MP of CD patients at 3 and 6 months post-denture insertion compared to 24 h post-insertion, regardless of the mandibular RR height [15]. This concurs with another study that reported that the longer the individuals use their dentures, the higher their MP suggesting that the duration of denture wearing was associated with MP [16]. However, the results of the ANCOVA (Table 5) showed that the duration of denture wearing had no significant impact on the MP of CD wearers. This finding can be attributed to the study's cross-sectional design, which did not examine the changes and improvements in MP after a series of follow-ups.

### 4.3 Study limitations

The sample size was one of our study's limitations. Because the participants were limited to hospital patients who provided their consent and could be evaluated, the gender distribution was uneven, and comparisons could not be made. The current study did not take into account the dentist's skills and expertise, denture quality [57], or the patient's oral conditions, such as RR resiliency [21], salivary flow rate [[25], [26]], tongue pressure [[10], [22], [24]], occlusal force [13,16,17], and maxillomandibular relationship [21], all of which could have influenced denture retention and stability during chewing. This study only conducted a one-time evaluation of the MP using test gummy jelly. The authors did not evaluate the MP subjectively, which could have led to different results [28]. These points must be considered in future studies to examine further the factors that affect the MP of CD wearers.

## 5 Conclusions

The MP of CD wearers varied, depending on the height and form of the RR, combined upper and lower RR morphology as well as the occlusal contact area of the CDs. Therefore, we believe that the underlying RR morphology and the occlusal contact status of the CDs are essential factors in predicting the treatment outcome of CD wearers.

## Ethics statement

This study was conducted after applying to and receiving approval from the Niigata University Ethics Committee (#28-R42-28).

## Author contributions

All authors contributed to the study's conceptualization and design. Ma. Therese Sta. Maria and Yoko Hasegawa wrote the first draft of the manuscript. Ma. Therese Sta. Maria, Yoko Hasegawa, Pinta Marito, Tasuku Yoshimoto and, Simonne Salazar performed the material preparation, data collection, and analysis. All authors contributed to the interpretation of the data. Takahiro Ono reviewed and edited the manuscript. All authors critically reviewed and commented on previous versions of the manuscript and agreed with the content of the final manuscript.

## Data availability statement

Data will be made available on request.

## Declaration of competing interest

The authors declare that they have no known competing financial interests or personal relationships that could have appeared to influence the work reported in this paper
